# Genetic influence on functional brain activation to an affective face matching task: an fMRI twin-study

**DOI:** 10.1038/s41398-026-04240-x

**Published:** 2026-07-09

**Authors:** Jörgen Rosén, Gránit Kastrati, Hampus Grönvall, Fara Tabrizi, Tomas Furmark, Fredrik Åhs

**Affiliations:** 1 Department of Clinical Neuroscience, Karolinska Institutet, Stockholm, Sweden; 2 Department of Education, Psychology and Social work, Mid Sweden University, Östersund, Sweden; 3 Department of Psychology, Uppsala University, Uppsala, Sweden

**Keywords:** Neuroscience, Diagnostic markers

## Abstract

Neural activations in response to pictures of faces have consistently been reported across a network of brain regions, including the lateral occipital face area, fusiform face area, superior temporal sulcus and the amygdala. Face-related activations in this network can be altered in anxiety conditions such as social anxiety disorder, but it is not known to what extent genetic factors influence the neural underpinnings of facial emotion recognition. We used the Hariri affective face matching task and functional magnetic resonance imaging to measure neural responses to negative (angry and fearful) facial-emotion categories in 64 pairs of monozygotic and 71 pairs of dizygotic healthy same sex twin pairs. Brain responses to negative faces were parcellated into 294 cytoarchitectonic regions according to the Jülich Brain Atlas. The variance in neural activation was then decomposed into additive genetic variance, common environmental factors, and specific environmental factors plus error measures (ACE model) in each region. The relationship between social anxiety, indexed by the Liebowitz Social Anxiety Scale (LSAS-SR), and brain activity was also assessed. Results were Bonferroni-corrected to control for false positives (p < 0.05). We found a small to moderate genetic influence on face-evoked neural responses in the amygdala, the lateral occipital face area, and the fusiform face area. There was a moderate genetic influence on social anxiety symptoms but individual differences in social anxiety did not predict brain activation. These findings demonstrate that genetic factors influence functional brain responses to faces expressing negative affect, as well as social anxiety traits, but the results do not provide evidence for shared genetic architecture between social anxiety symptomatology and neural responsiveness to negative facial emotions.

## Introduction

Face viewing paradigms have been used extensively in neuroimaging studies of emotions and psychiatric disorders. One of the most widely used tasks is the Hariri emotional face-shape matching task [1], which was developed to probe amygdala activation. Individual differences in amygdala activation to threatening social signals, such as negative emotional expressions in the case of this task, are thought to inform on the neurobiology of anxiety and depressive symptoms [2]. Of all anxiety disorders, especially social anxiety disorder (SAD) could be hypothesized to involve altered amygdala reactivity to faces due to biased processing of socio-emotional signals [3]. Neuroimaging studies have reported exaggerated amygdala activation to emotional faces in SAD [4, 5] and attenuated activation after successful treatment [6]. The disorder is moderately heritable according to a meta-analysis of twin-studies [7].

Initial studies of genetic influences on face-related amygdala activation used a candidate gene approach [8] and reported that variation in the promoter region of the serotonin transporter gene (5-HTTLPR) affected amygdala activation in healthy volunteers [1] as well as in SAD patients [9]. Later meta-analyses, however, showed that this effect was negligible or non-existent [10]. So far, genome-wide association studies (GWAS) have failed to demonstrate significant and replicable effects of variation in single-nucleotide polymorphisms on brain function in the Hariri emotional face-shape matching task in a non-clinical sample [11]. A study of SAD, using a family design, however suggested that amygdala hyperreactivity to faces is at least moderately heritable [12]. To our knowledge, twin studies have not yet investigated genetic influences on neural activity evoked by this task.

Twin studies estimate additive genetic influences on phenotypes by comparing within pair correlations between monozygotic twins, who share a 100% of their genetic material, with dizygotic twins, who share 50%. Twin methodology has successfully been used to estimate the heritability of the volume of different brain areas [13]. Regarding the amygdala, the genetic influence on regional brain volume is between 60% and 80% [14]. A handful of studies have investigated genetic influences on task-related brain function using a twin design [15–17]. These studies have shown that small to moderate genetic influences on brain function are detectable in sample sizes of 200–400 twins. The reported genetic influences are strongest in brain areas that are most activated by the task. This could reflect poor test-retest reliability in areas with low task related signal, reducing estimates of additive genetic effects [18]. Indeed, the reliability of fMRI responses to face-matching tasks has been found to be low [19, 20]. Improving the precision of task-based fMRI measurements is therefore a priority and can be achieved by averaging the signal from multiple voxels within a region [21]. We therefore parcellated the brain into regions consisting of multiple voxels before estimating genetic influences on face related activity.

**Fig. 1:**
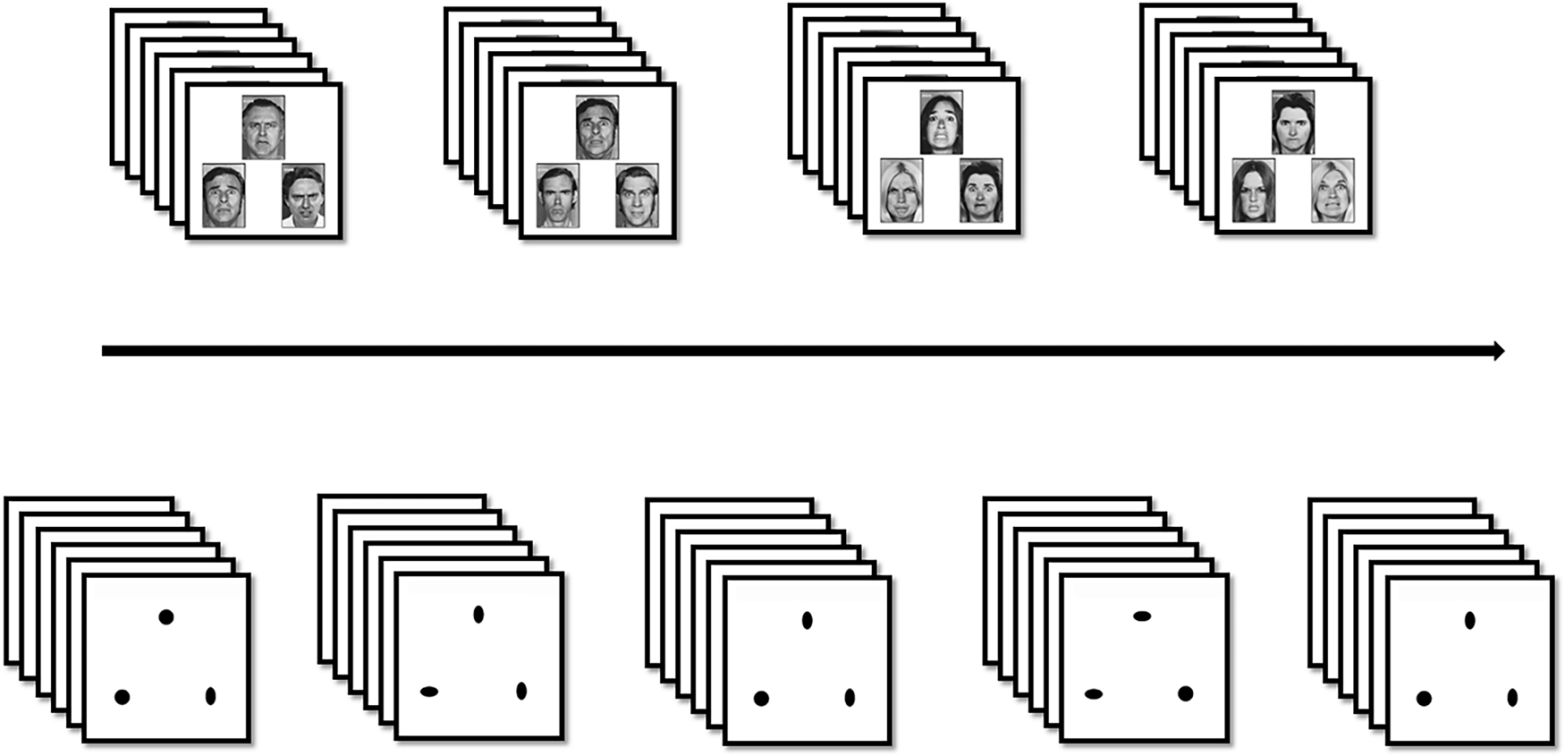
The experimental fMRI paradigm. This consisted of 4 blocks of an affective face-processing task interleaved with 5 blocks of a sensorimotor control task.

We here measured brain function during the Hariri emotional face-shape matching task (Fig. 1) using functional magnetic resonance imaging (fMRI). By comparing brain reactivity to angry and fearful faces relative to shapes between monozygotic and dizygotic twin pairs, we estimated the additive genetic contribution to the targeted brain function. We selected this widely used paradigm because it is a well-validated probe of threat-related face processing, facilitating comparability with a large body of prior work (including twin and social-anxiety studies) showing robust amygdala and fusiform responsivity to negatively valenced facial expressions. Accordingly, our a priori hypotheses focused on neural reactivity to socially salient threat cues (anger/fear), rather than on effects driven by positive valence. The shape-matching condition served as an active baseline that controlled for visuospatial matching and motor/attentional demands while minimizing social-emotional content. Apart from the amygdala, the face task is also known to activate the lateral occipital face area, the superior temporal sulcus, and the fusiform face area [1]. We hypothesized that there would be a genetic contribution to face reactivity in these regions. We further tested the prediction that there would be a correlation between individual differences in social anxiety symptoms and amygdala activation, as suggested by previous research [5], and that this correlation may be attributable to shared genetic influence. Thus, we also estimated the genetic influence on social anxiety symptoms in our twin sample.

## Method

### Subjects

During 2017–2018, same-sex twins between the ages of 20 and 60 were recruited through the Swedish Twin Registry. A total of 3021 individuals were invited to participate in the study by mail, and of those, 646 applicants signed up for participation. Participants were excluded if they were unable to undergo an MRI examination safely because of medical implants or other non-removable metal in the body, ongoing substance abuse, ongoing psychological treatment and/or use of medication affecting emotion or cognition. After the initial screening, 305 participants underwent fMRI. After data collection, participants were excluded because of excessive head motion (3), inadequate performance on the experimental task (10), or missing data or an incomplete twin pair (22). The final sample of 270 twins included 64 MZ pairs (38 female and 26 male pairs) and 71 DZ pairs (40 female and 31 male pairs) mean age 33.27 years (SD = 10.21, age range 20–58) (see Supplementary Table 1 for demographic characteristics of the twin sample). Participants received SEK 1000 (roughly equivalent to 100 USD) as reimbursement for their participation. All participants included in the study provided written informed consent. The study was approved by the Uppsala Ethical Review Board (Dnr 2014-01160) and was conducted in accordance with the relevant guidelines and regulations outlined by this review board.

### Materials

#### Stimulus presentation software

Stimuli were presented on a flat screen in the MR scanner with the help of projector (Epson EX5260). The computer running the stimulus presentation used a custom version of Unity (version 5.2.3, Unity Technologies, San Francisco, CA) and communicated with BIOPAC (BIOPAC Systems, Goleta, CA) through a parallel port interface. The software for the parallel port interface was custom made and used standard .NET serial communication libraries by Microsoft (Microsoft Corporation, Albuquerque, New Mexico). Stimulus presentation software can be obtained upon request.

### Brain imaging

Imaging data were acquired using a 3.0 T scanner (Discovery MR750, GE Healthcare) and an 8-channel head-coil at Karolinska Institute MRI Center Core Facility in Stockholm, Sweden. Foam wedges, earplugs and headphones were used to reduce head motion and scanner noise. T1-weighted structural images were acquired with whole-head coverage, TR = 6400 ms, TE = 28 ms, acquisition time 6.04 min and flip angle 11 (degrees). Functional images were acquired using gradient echo-planar-imaging (EPI), TR = 2390 ms, TE = 28 ms, flip angle = 80 (degrees), slice thickness 3.0 mm with no spacing, axial orientation, frequency direction R/L, interleaved bottom up. Higher order shimming was performed and the number of dummy scans before the experiment was five. Total number of slices for every acquired volume was 47 with an isotropic voxel size of 3.0 mm.

### Liebowitz social anxiety scale

Social anxiety symptom severity was measured with the Liebowitz Social Anxiety Scale, LSAS-SR (Swedish version) [22]. The LSAS-SR yields a total score ranging from 0 to 144, with higher scores indicating greater symptom severity.

### Procedure

#### Face-matching task

The experimental fMRI paradigm (Fig. 1) consisted of 4 blocks of a face-processing task interleaved with 5 blocks of a sensorimotor control task [1, 23]. Participant performance (accuracy and reaction time) was monitored during all scans using an MR-compatible button box. During the face-processing task, participants viewed a trio of faces with angry or fearful expressions and selected 1 of 2 faces (bottom) identical to a target face (top). We focused on fearful/angry expressions because this task variant is a widely used and well-validated probe of threat-related face processing, and because our a priori hypotheses concerned reactivity to socially salient threat cues. Each face-processing block consisted of 6 images of the same expression, balanced for gender, all of which were derived from a standard set of pictures of facial affect [24]. The order of the face-processing blocks was the same across all participants to ensure that twin pairs performed the tasks in the same sequence. During the sensorimotor control blocks, participants viewed a trio of simple geometric shapes (circles and ellipses) and selected 1 of 2 shapes (bottom) identical to a target shape (top). The shape condition served as an active baseline controlling for visuospatial matching and motor/attentional demands while minimizing social-emotional content. Each sensorimotor control block consisted of 6 different shape trios. All blocks were preceded by a brief instruction (“Match faces” or “Match shapes”) that lasted 2 s. In the face-processing blocks, each of the 6 face trios was presented for 4 s with a variable interstimulus interval (ISI) of 2 to 6 s (mean, 4 s), for a total block length of 48 s. A variable ISI was used to minimize expectancy effects and resulting habituation and to maximize amygdala reactivity throughout the paradigm. In the sensorimotor control blocks, each of the 6 shape trios was presented for 4 s with a fixed inter-stimulus interval of 2 s, for a total block length of 36 s. Total task length was 390 s. Apart from the face-matching task, participants also performed an interpersonal distance task and a fear conditioning task. Data from these tasks have been reported separately [16, 17, 25, 26].

### Statistical analysis of imaging data

Analyses of fMRI-data were performed using SPM12 (Wellcome Department of Cognitive Neurology, University College, London) implemented in Matlab 2019a (MathWorks, Inc., Natick, MA). Preprocessing of images were performed using interleaved slice time correction and included re-alignment and co-registration to the anatomical T1-weighted image. Images were spatially normalized to Montreal Neurological Institute (MNI) standard space. Normalized images were smoothed with an 8 mm FWHM Gaussian kernel.

Statistical analysis included a voxel based first-level analysis and a region of interest (ROI) based second-level analysis. First-level analysis used event-related modeling in the SPM12 software. Trial onsets for the faces and shapes were modeled as two separate regressors with a trial duration of 4–8 s. Realignment parameters were entered as six separate regressors to correct for head movements during image acquisition (rotation x, y z and direction x, y, z). Regressors were convolved with a hemodynamic response function. Contrast images for each participant were computed for the face matching task with faces greater than shapes.

In the group-level analysis we used simple t-tests testing whether the contrast estimate for each ROI was different from zero. Mean contrast estimates for every ROI were extracted with the help of the spm_read_vols.m function implemented in SPM12. We then performed t-tests on all extracted ROI estimates in R (http://www.R-project.org/) [27]. The level of significance was set to p < 0.05 (Bonferroni-corrected). We corrected for a total of 296 comparisons (the number of included ROIs in the analysis) to minimize the rate of false positives.

To assess brain–behavior associations, we correlated LSAS-SR total scores with extracted ROI contrast estimates (faces > shapes). Correlations were computed using Pearson’s *r* (two-tailed), and results are reported in the Results section.

Extraction of mean contrast estimates was performed using the Julich Brain Atlas implemented in SPM Anatomy toolbox v.2.2c [28], which is based on cytoarchitectonic differences between brain areas. The choice of using the cytoarchitectonic Juelich Brain Atlas for parcellation of contrast images was based on previous findings showing that gene expression differs along a posterior-anterior gradient in the visual stream across these regions [21]. The Julich Brain Atlas v29 contains 296 cytoarchitectonic masks. All ROIs were used. The choice of ROIs was therefore unbiased, as ROI definitions were independent of our study.

### Sensitivity analysis

We performed statistical analyses to evaluate whether results could be attributed to differences between male and female participants. To evaluate if responses differed between men and women, we performed two sample t-test with Bonferroni-correction.

### Estimation of genetic influences on brain function

#### Identification of outliers

Before the genetic modeling, we identified univariate outliers in our sample to increase the robustness of the estimated correlations between MZ and DZ twins. Removal of outliers is recommended in samples of fewer than 200 individuals [29] and has previously been used in a neuroimaging study of twins performing a working memory task [30]. Identification and exclusion of outliers were performed for each ROI before the analysis. We visualized outliers using boxplots in R and removed any participant with a mean contrast estimate deviating more than 1.5 times the interquartile range above the upper quartile or below the lower quartile. If one twin in a twin-pair was categorized as outlier, the co-twin was also excluded from the statistical analysis.

#### Estimation of genetic effects

After outliers had been excluded, additive genetic influences of brain data and SCR were estimated using the Mets software package [31, 32] implemented in R. We modeled data by decomposing sources of variation in contrast estimates from the analysis of fMRI data into additive genetic factors (A), common environmental factors (C) and non-shared environmental and error factors (E). The A, C, and E factors were estimated by contrasting MZ twin-pair correlations with DZ twin-pair correlations. The A factor can be identified because MZ twins are genetically identical whereas DZ-twins share 50% of their co-segregating alleles. Additionally, we assume that a shared environmental contribution (C) is equally shared within pairs regardless of whether they are MZ or DZ twins. Finally, unique variance is estimated as an E-factor and represents unique individual experiences and error. The A, C and E factors were estimated for each of the 296 ROIs for the contrast faces over shapes.

## Results

### Genetic influence on neural responses to the face-matching task

Variance in neural responses to the face-matching task was divided into additive genetic (*A*), common environmental (*C*) and unique environmental (*E*) factors (ACE-model). Overall, we found a statistically significant genetic influence on face related activation in 43 out of the 294 (15%) ROIs analyzed.

To increase the robustness of our analysis of amygdala activation, we constructed one left and one right amygdala ROI by aggregating across all atlas-defined amygdala subnuclei in the Jülich Brain Atlas (i.e., the full set of subregions that together comprise the atlas-defined amygdala in each hemisphere). Specifically, we first extracted participant-level contrast estimates (faces > shapes) separately for each amygdala subregion, and then computed a single hemisphere-level amygdala estimate per participant as an atlas/volume-weighted average across the included subregions. ACE models were then fit to these hemisphere-level (left/right) amygdala ROI estimates. Using these combined ROIs, we observed a small but statistically significant genetic influence in both the left and right amygdala (left amygdala: MZ n = 58, DZ n = 64, MZ *r* = 0.19, DZ *r* = 0.10, *A* = 0.19 (*p* < 0.05), *C* = 0.00 (*p* = 1), *E* = 0.81 (*p* < 0.05); right amygdala: MZ n = 57, DZ n = 64, MZ *r* = 0.16, DZ *r* = 0.08, *A* = 0.16 (*p* < 0.05), *C* = 0.00 (*p* = 1), *E* = 0.84 (*p* < 0.05)). To avoid implying that multiple subregions showed significant heritability, we additionally report ACE results for each amygdala subregion separately in the Supplementary Table 3 and note that the hemisphere-level estimate was primarily driven by the ventromedial amygdala subregion (VTM), whereas the remaining amygdala subregions did not show statistically significant genetic influence after correction.

When considering brain regions previously discussed as face processing areas, we found small to moderate genetic influences (*A*) on face related responses in the lateral occipital face area (Visual h0c4v) and the fusiform face area (FG2 and FG4). We also found genetic influences on activation in the dorsal visual cortex as well as in parietal areas.

**Fig. 2:**
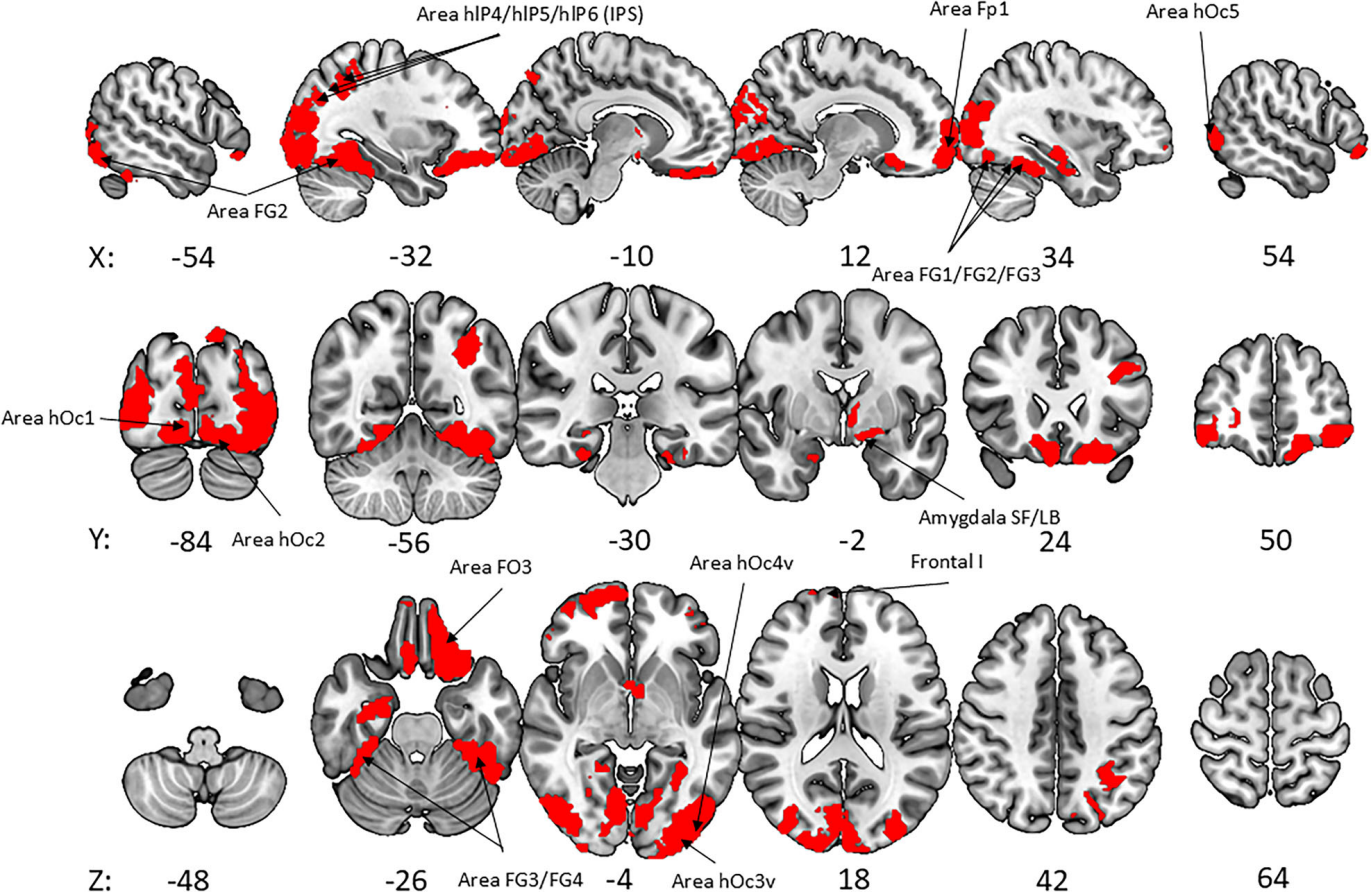
Genetic influences on face-evoked brain responses. Areas in red denote brain regions from the Jülich brain atlas (v29) with statistically significant genetic influence on brain responses for emotional faces over shapes. All areas displayed are Bonferroni-corrected at *p* < 0.05. FO fronto orbital, FG fusiform gyrus, FP frontal pole, SF/LB superficial and laterobasal amygdala group, IPS intraparietal sulcus, hOc1/2/5 anterior occipital sulcus. hOc3/4v lateral occipital face area.

We also noted small to moderate genetic influence also in the basal forebrain, the orbitofrontal cortex, the frontal pole, and the motor cortex. In temporal cortices, a genetic influence was observed in the auditory cortex, hippocampus (HATA, DG and CA1/CA2), and insula. Activation in several thalamic regions was also influenced genetically. See Table 1 and Fig. 2.

**Table 1:** Brain regions in which genetic influences on brain function were noted (at p < 0.05, Bonferroni corrected) for faces over shapes.

Region	MZ-pairs	DZ-pairs	MZ_r_	DZ_r_	A	A 95CI lower	A 95CI upper	C	C 95CI lower	C 95CI upper	E	E 95CI lower	E 95CI upper
Area FG3 (FusG) right	60	68	0.45	0.22	0.45	0.25	0.64	0.00	0.00	0.00	0.55	0.36	0.75
Ch 123 (Basal Forebrain) right	56	63	0.44	0.22	0.44	0.20	0.68	0.00	0.00	0.00	0.56	0.32	0.80
Area hOc4v (LingG) left	61	69	0.43	0.22	0.43	0.26	0.61	0.00	0.00	0.00	0.57	0.39	0.74
Area hIP5 (IPS) left	55	70	0.43	0.21	0.43	0.18	0.68	0.00	0.00	0.00	0.57	0.32	0.82
Ch 123 (Basal Forebrain) left	58	62	0.42	0.21	0.42	0.20	0.63	0.00	0.00	0.00	0.58	0.37	0.80
Area Fo6 (OFC) left	53	69	0.41	0.20	0.41	0.17	0.65	0.00	0.00	0.00	0.59	0.35	0.83
Area hIP4 (IPS) left	56	69	0.40	0.20	0.40	0.14	0.65	0.00	0.00	0.00	0.60	0.35	0.86
Ch 4 (Basal Forebrain) left	59	60	0.39	0.19	0.39	0.20	0.58	0.00	0.00	0.00	0.61	0.42	0.80
Area hOc5 (LOC) right	60	69	0.39	0.19	0.39	0.19	0.58	0.00	0.00	0.00	0.61	0.42	0.81
Area hOc4la (LOC) left	61	68	0.38	0.19	0.38	0.21	0.56	0.00	0.00	0.00	0.62	0.44	0.79
Area hIP1 (IPS) left	57	69	0.37	0.19	0.37	0.16	0.59	0.00	0.00	0.00	0.63	0.41	0.84
Area Fo5 (OFC) left	55	66	0.37	0.18	0.37	0.15	0.59	0.00	0.00	0.00	0.63	0.41	0.85
Area hOc2 (V2 18) left	59	65	0.37	0.18	0.37	0.19	0.55	0.00	0.00	0.00	0.63	0.45	0.81
CA2 (Hippocampus) right	58	64	0.37	0.18	0.37	0.16	0.57	0.00	0.00	0.00	0.63	0.43	0.84
Area hOc3d (Cuneus) right	57	63	0.37	0.18	0.37	0.17	0.56	0.00	0.00	0.00	0.63	0.44	0.83
CA1 (Hippocampus) right	58	62	0.36	0.18	0.36	0.16	0.57	0.00	0.00	0.00	0.64	0.43	0.84
Area FG4 (FusG) left	59	71	0.35	0.18	0.35	0.16	0.55	0.00	0.00	0.00	0.65	0.45	0.84
Area Fo5 (OFC) right	58	70	0.35	0.17	0.35	0.14	0.56	0.00	0.00	0.00	0.65	0.44	0.86
Area 7PC (SPL) left	57	65	0.34	0.17	0.34	0.13	0.56	0.00	0.00	0.00	0.66	0.44	0.87
Area hOc4la (LOC) right	59	67	0.34	0.17	0.34	0.11	0.57	0.00	0.00	0.00	0.66	0.43	0.89
Area Fo2 (OFC) right	56	64	0.33	0.16	0.33	0.08	0.57	0.00	0.00	0.00	0.67	0.43	0.92
Area Fo3 (OFC) left	57	65	0.32	0.16	0.32	0.10	0.54	0.00	0.00	0.00	0.68	0.46	0.90
Area hOc3v (LingG) left	55	71	0.32	0.16	0.32	0.06	0.57	0.00	0.00	0.00	0.68	0.43	0.94
Area hOc4lp (LOC) left	57	68	0.31	0.16	0.31	0.11	0.52	0.00	0.00	0.00	0.69	0.48	0.89
Area Fo6 (OFC) right	54	70	0.29	0.15	0.29	0.02	0.57	0.00	0.00	0.00	0.71	0.43	0.98
Area hIP4 (IPS) right	60	68	0.29	0.14	0.29	0.09	0.48	0.00	0.00	0.00	0.71	0.52	0.91
Area Fp1 (FPole) right	53	67	0.29	0.14	0.29	0.01	0.56	0.00	0.00	0.00	0.71	0.44	0.99
Area Fo7 (OFC) left	55	65	0.28	0.14	0.28	0.06	0.51	0.00	0.00	0.00	0.72	0.49	0.94
HATA (Hippocampus) right	60	65	0.27	0.14	0.27	0.05	0.50	0.00	0.00	0.00	0.73	0.50	0.95
Area hOc5 (LOC) left	60	69	0.27	0.13	0.27	0.07	0.47	0.00	0.00	0.00	0.73	0.53	0.93
Area hOc4lp (LOC) right	58	64	0.26	0.13	0.26	0.06	0.47	0.00	0.00	0.00	0.74	0.53	0.94
Area FG2 (FusG) left	57	66	0.25	0.13	0.25	0.04	0.47	0.00	0.00	0.00	0.75	0.53	0.96
Area FG1 (FusG) right	60	66	0.25	0.12	0.25	0.02	0.47	0.00	0.00	0.00	0.75	0.53	0.98
VTM (Amygdala) left	55	63	0.24	0.12	0.24	0.01	0.48	0.00	0.00	0.00	0.76	0.52	0.99
Area Ph3 (PhG) right	61	64	0.24	0.12	0.24	0.02	0.46	0.00	0.00	0.00	0.76	0.54	0.98
Area IFS3 (IFS) left	57	62	0.24	0.12	0.24	0.02	0.46	0.00	0.00	0.00	0.76	0.54	0.98
HC Subiculum (Hippocampus) right	57	61	0.24	0.12	0.24	0.03	0.45	0.00	0.00	0.00	0.76	0.55	0.97
Area hOc2 (V2 18) right	59	65	0.24	0.12	0.24	0.03	0.44	0.00	0.00	0.00	0.76	0.56	0.97
HC Prosubiculum (Hippocampus) right	57	64	0.23	0.12	0.23	0.01	0.46	0.00	0.00	0.00	0.77	0.54	0.99
Area FG3 (FusG) left	60	69	0.23	0.12	0.23	0.01	0.45	0.00	0.00	0.00	0.77	0.55	0.99
BST (Bed Nucleus) left	58	62	0.23	0.11	0.23	0.02	0.43	0.00	0.00	0.00	0.77	0.57	0.98
Area hIP8 (IPS) left	60	68	0.22	0.11	0.22	0.01	0.44	0.00	0.00	0.00	0.78	0.56	0.99
Area IFS1 (IFS) left	60	63	0.22	0.11	0.22	0.02	0.43	0.00	0.00	0.00	0.78	0.57	0.98

Additive genetic influence (A), common environmental factors (C) and unique environmental factors (E) together with correlation coefficients for monozygotic (MZ r) and dizygotic (DZ r) twins are tabulated. Columns with MZ- and DZ-pairs show the total number of twin pairs included for each analysis. The number of twin pairs differs between regions because outliers were removed prior to the estimation of the twin model.

**Fig. 3:**
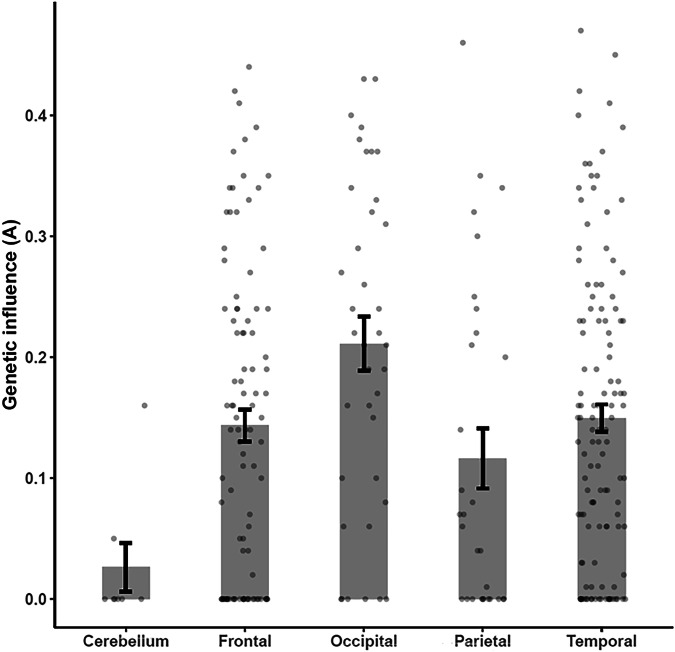
Bar graphs depicting mean genetic influence (A) on face-related responses in brain regions included in the Jülich brain atlas that were partitioned into cerebellum, frontal, occipital, parietal and temporal areas. Dots represent the contribution of genetic influence (A) from each unique region of the Jülich brain atlas. Error bars show standard error.

To better understand in which brain lobes the genetic influence was highest, we measured mean genetic influence in ROIs that were part of the cerebellum, frontal, occipital, parietal, and temporal areas (see Fig. 3). We found that occipital regions (*F* = 4.53, *p* < 0.05, mean *A* = 0.23) had higher mean genetic influence compared to parietal areas (*t* = 3.01, p < 0.05, mean *A* = 0.13) and the cerebellum (*t* = 3.70, p < 0.05, mean *A* = 0.03).

### Genetic influence on self-reported social anxiety

An ACE model was used to estimate genetic and environmental effects on self-reported social anxiety (LSAS-SR total score) in the same manner as for ROI data. In this community sample, LSAS-SR scores showed variability across participants (mean = 34.86, SD = 23.16, min = 0, max = 115, observed mean and dispersion are provided in Supplementary Table 1). There was a moderate statistically significant genetic influence on LSAS-SR score (MZ *r* = 0.54, DZ *r* = 0.30, MZ n = 60, DZ n = 70, *A* = 0.46 (*p* < 0.05), *C* = 0.08 (*p* = 0.46), *E* = 0.45 (*p* < 0.05)). We assessed brain–behavior associations by correlating LSAS-SR total scores with extracted ROI contrast estimates (Pearson’s *r*, two-tailed) but found no statistically significant correlations.

### Group level analysis of fMRI responses to the face-matching task

Statistically significant brain responses to the face-matching task were observed across a wide range of brain areas, including the amygdala, the fusiform face area (FG2 and FG4), visual areas hOc3v, hOc4v (lateral occipital face areas), the thalamus and the hippocampus. Also, in the prefrontal cortex, the orbitofrontal cortex (OFC Fo3) and inferior frontal cortex (BA44 and BA45), increased responses to faces relative to shapes were observed. See Supplementary Table 3 for a summary of ROI-based results. For comparison, we also include results from the voxel-based analysis in Supplementary Table 5. To control for potential confounds based on participants’ sex, we compared brain responses to the face-matching task between men and women. No statistically significant difference between men and women was found (highest *t* = 3.8, *p* = 0.07 in right amygdala).

## Discussion

We estimated the genetic influence on brain responses to negative faces assessed with the Hariri affective face-matching task using the twin method (monozygotic n = 64 pairs, dizygotic n = 71 pairs). A small genetic influence on activation to faces was observed in the amygdala, whereas moderate genetic influences were found on activation in the lateral occipital face area, the fusiform face area, the hippocampus, the insula, and the dorsal visual cortex, as well as in parietal and forebrain areas. A moderate genetic influence was also observed for self-reported measures of social anxiety, but we found no correlation between face-evoked brain function and symptoms of social anxiety. This pattern suggests that, within a community twin sample, genetic factors contribute to both social anxiety symptoms and neural reactivity to negative facial expressions, but do not provide evidence for a shared genetic architecture between these measures.

The Hariri faces-shapes matching task was designed to elicit robust responses in core face- and threat-processing circuitry, including the amygdala. We noted a moderate genetic influence (*A* = 0.46) on self-reported symptoms of social anxiety which is comparable to twin estimates of heritability of SAD (*A* = 0.42) in much larger samples [33]. We also observed a small but statistically significant additive genetic influence on amygdala responses evoked by negatively valenced faces. Importantly, however, we did not observe an association between amygdala activation and social anxiety symptoms in this sample. Therefore, our findings should be interpreted as evidence that reactivity to negative facial expressions constitutes a modestly heritable neural phenotype, rather than as support for face-reactivity as a biomarker or endophenotype of social anxiety. Establishing an endophenotype/biomarker for SAD would require additional evidence (e.g., robust symptom or diagnosis associations, and replication in clinically diagnosed samples), which was beyond the scope of the present study.

Our finding of a moderate genetic influence on neural responses to social stimuli in the occipital face area and the fusiform face area is in line with previous reports of greater correlations in face related responses in identical than fraternal twins in these areas [15, 34]. These brain areas are also the regions where the strongest activations to facial stimuli were observed, suggesting a relation between the magnitude of genetic influence and the magnitude of the task-evoked response. Previous twin studies have demonstrated a genetic influence on task-related fMRI responses in the range of 20–60%, depending on the task [16, 17, 25, 30]. Supporting the idea of a relation between the magnitude of the response and the genetic influence on the response, the highest genetic influence has been found in areas activated by the task. In Blokland et al. [30], the strongest genetic influences on activation were found in the lateral prefrontal cortex during a working memory task. In Kastrati et al. [16] the largest genetic effects were observed in the insula and thalamus during a fear conditioning task, consistent with strong activation to the conditioned fear cue relative to the control cue in these areas. Further, during an inter-personal distance task, the largest genetic influences were found on activation in the occipital cortices which was also the brain area where the greatest task activations were found (Rosén et al. [17]). These reports show that genetic influences reliably can be observed in the set of neural regions specifically activated by a task using twin methods. However, brain-behavior relationships could be different in clinical populations.

Moreover, the integration of our functional activation data with the morphometric findings of Abbasi et al. [15] highlights the importance of considering both brain structure and function when investigating the neurogenetic basis of social cognition. The parallel genetic effects observed in these studies suggest that heritable factors may underlie not only the physical architecture of face-selective regions but also their dynamic responses to affective stimuli. This has important implications for understanding the biological mechanisms that contribute to individual differences in social perception and related psychiatric conditions.

There are several limitations to the present study. The number of monozygotic and dizygotic twin pairs in our study was similar to that in other neuroimaging studies in twins, but it is still low for a twin study. To ensure that additive genetic effects were not driven by outliers, all outliers were removed before estimation of the twin models. We also used an ROI approach, instead of voxel-wise analyses, to increase the level of signal relative to noise ratio in the task-related fMRI data. It should also be noted that the test-retest reliability of task-based fMRI data is generally low [19]. More specifically, the test-retest reliability of the Hariri face-matching task had an intra-class correlation coefficient of 0.2–0.4 depending on region, in a study that used data from the UK Biobank [35]. It should be noted that the additive genetic influence is dependent on the measurement reliability and therefore might have limited the scale of our estimates of additive genetic influences on brain function.

In conclusion, results indicate modest genetic influences on task-evoked brain responses to negatively valenced facial expressions in the amygdala, occipital face area and fusiform face area, as well as a moderate genetic influence on dimensional social anxiety symptoms, in a healthy mono- and dizygotic twin sample. Although we did not observe symptom–brain activation associations in this cohort, these findings provide a basis for future studies to test whether these heritable neural responses relate to clinically diagnosed social anxiety disorder and other anxiety phenotypes.

## Supplementary information

Supplemental material

## Data Availability

All data generated during the current study can be made available upon reasonable request from the corresponding author.
