# Supplementary material for: Genetic influence on functional brain activation to an affective face matching task: an fMRI twin-study
Source: Transl Psychiatry. 2026 Jul 9;16:351. doi: 10.1038/s41398-026-04240-x (PMC13351005; doi:10.1038/s41398-026-04240-x)

**Supplementary materials**

**Supplementary Table 1.** Demographic characteristics of the twin sample. Educational level was rated 0-3 (lowest-highest). We found no statistically significant mean difference for educational level or self-reported measures of social anxiety (LSAS-SR) between co-twins or by zygosity. The sample included 246 right and 27 left-handed participants (with 16 participants missing data). MZ = monozygotic twins, DZ = dizygotic twins

|  | MZ | DZ | Total |
| --- | --- | --- | --- |
|  | (n = 128) | (n = 142) | (n = 270) |
| Age (mean ± SD) | 33.82 (8.28) | 32.78 (11.69) | 33.27 (10.21) |
| Sex (n = M/F) | 52 / 76 | 62 / 80 | 122 / 172 |
| Educational level (mean ± SD) | 2.70 (0.77) | 2.66 (0.63) | 2.68 (0.70) |
| LSAS-SR | 35.52 (22.76) | 34.26 (23.59) | 34.86 (23.16) |

**Supplementary Table 2.** Brain regions with genetic influence on brain function surviving the statistical criterion (p < 0.05, Bonferroni corrected) for shapes over faces. Additive genetic influence (A), common environment (C) and unique environment (E) together with correlation coefficients for monozygotic (MZ r) and dizygotic (DZ r) twins are tabulated. Columns with MZ- and DZ-pairs show the total number of twin pairs included for each analysis.

| Region | *MZ-pairs* | *DZ-pairs* | *MZ_r_* | *DZ_r_* | *A* | *A 95CI lower* | *A 95CI upper* | *C* | *C 95CI lower* | *C 95CI upper* | *E* | *E 95CI lower* | *E 95CI upper* |
| --- | --- | --- | --- | --- | --- | --- | --- | --- | --- | --- | --- | --- | --- |
| Area TE 1.2 (HESCHL) right | 57 | 69 | 0.47 | 0.24 | 0.47 | 0.27 | 0.67 | 0.00 | 0.00 | 0.00 | 0.53 | 0.33 | 0.73 |
| Area 2 (PostCS) right | 57 | 65 | 0.46 | 0.23 | 0.46 | 0.22 | 0.70 | 0.00 | 0.00 | 0.00 | 0.54 | 0.30 | 0.78 |
| Area Id1 (Insula) right | 58 | 69 | 0.42 | 0.21 | 0.42 | 0.21 | 0.63 | 0.00 | 0.00 | 0.00 | 0.58 | 0.37 | 0.79 |
| Area PFop (IPL) right | 57 | 69 | 0.40 | 0.20 | 0.40 | 0.17 | 0.63 | 0.00 | 0.00 | 0.00 | 0.60 | 0.37 | 0.83 |
| Area PFt (IPL) right | 59 | 64 | 0.36 | 0.18 | 0.36 | 0.13 | 0.58 | 0.00 | 0.00 | 0.00 | 0.64 | 0.42 | 0.87 |
| Area 3b (PostCG) right | 56 | 68 | 0.35 | 0.18 | 0.35 | 0.10 | 0.60 | 0.00 | 0.00 | 0.00 | 0.65 | 0.40 | 0.90 |
| Area Ph2 (PhG) right | 62 | 63 | 0.35 | 0.17 | 0.35 | 0.15 | 0.55 | 0.00 | 0.00 | 0.00 | 0.65 | 0.45 | 0.85 |
| Area TE 2.1 (STG) right | 57 | 67 | 0.34 | 0.17 | 0.34 | 0.09 | 0.60 | 0.00 | 0.00 | 0.00 | 0.66 | 0.40 | 0.91 |
| Area OP4 (POperc) right | 58 | 65 | 0.34 | 0.17 | 0.34 | 0.13 | 0.55 | 0.00 | 0.00 | 0.00 | 0.66 | 0.45 | 0.87 |
| Area OP1 (POperc) right | 59 | 69 | 0.34 | 0.17 | 0.34 | 0.12 | 0.55 | 0.00 | 0.00 | 0.00 | 0.66 | 0.45 | 0.88 |
| Area Ph2 (PhG) left | 58 | 69 | 0.33 | 0.16 | 0.33 | 0.08 | 0.57 | 0.00 | 0.00 | 0.00 | 0.67 | 0.43 | 0.92 |
| DG (Hippocampus) right | 58 | 68 | 0.33 | 0.16 | 0.33 | 0.09 | 0.56 | 0.00 | 0.00 | 0.00 | 0.67 | 0.44 | 0.91 |
| Area 5Ci (SPL) right | 59 | 59 | 0.32 | 0.16 | 0.32 | 0.13 | 0.52 | 0.00 | 0.00 | 0.00 | 0.68 | 0.48 | 0.87 |
| Area 8d2 (SFG) left | 52 | 61 | 0.32 | 0.16 | 0.32 | 0.10 | 0.54 | 0.00 | 0.00 | 0.00 | 0.68 | 0.46 | 0.90 |
| Area TE 2.2 (STG) right | 58 | 64 | 0.31 | 0.15 | 0.31 | 0.10 | 0.52 | 0.00 | 0.00 | 0.00 | 0.69 | 0.48 | 0.90 |
| Area 3a (PostCG) right | 55 | 66 | 0.30 | 0.15 | 0.30 | 0.06 | 0.54 | 0.00 | 0.00 | 0.00 | 0.70 | 0.46 | 0.94 |
| Area TE 1.0 (HESCHL) right | 57 | 71 | 0.29 | 0.15 | 0.29 | 0.03 | 0.56 | 0.00 | 0.00 | 0.00 | 0.71 | 0.44 | 0.97 |
| Area TE 1.2 (HESCHL) left | 58 | 65 | 0.28 | 0.14 | 0.28 | 0.05 | 0.51 | 0.00 | 0.00 | 0.00 | 0.72 | 0.49 | 0.95 |
| Area Id1 (Insula) left | 54 | 62 | 0.26 | 0.13 | 0.26 | 0.02 | 0.50 | 0.00 | 0.00 | 0.00 | 0.74 | 0.50 | 0.98 |
| Area TE 1.1 (HESCHL) right | 58 | 69 | 0.26 | 0.13 | 0.26 | 0.04 | 0.48 | 0.00 | 0.00 | 0.00 | 0.74 | 0.52 | 0.96 |
| Area STS2 (STS) right | 56 | 66 | 0.26 | 0.13 | 0.26 | 0.01 | 0.50 | 0.00 | 0.00 | 0.00 | 0.74 | 0.50 | 0.99 |
| Area PFm (IPL) left | 58 | 65 | 0.23 | 0.12 | 0.23 | 0.02 | 0.45 | 0.00 | 0.00 | 0.00 | 0.77 | 0.55 | 0.98 |
| Area TE 3 (STG) left | 56 | 61 | 0.23 | 0.11 | 0.23 | 0.01 | 0.45 | 0.00 | 0.00 | 0.00 | 0.77 | 0.55 | 0.99 |

**Supplementary Table 3.** Brain regions with higher responses to faces over shapes (*p* < 0.05, Bonferroni corrected).

| *Region* | *t_269_* | *-log_10_ p* | *Contrast estimate* | *95 CI lower* | *95 CI upper* |
| --- | --- | --- | --- | --- | --- |
| Area hOc3v (LingG) left | 34.49 | 97.54 | 0.81 | 0.76 | 0.85 |
| Area hOc4v (LingG) right | 34.23 | 96.83 | 1.16 | 1.09 | 1.22 |
| Area hOc4v (LingG) left | 33.32 | 94.28 | 1.04 | 0.98 | 1.10 |
| Area hOc3v (LingG) right | 32.31 | 91.41 | 0.84 | 0.79 | 0.89 |
| Area FG4 (FusG) right | 31.12 | 87.95 | 0.62 | 0.58 | 0.65 |
| Area IFS2 (IFS) right | 28.92 | 81.33 | 0.93 | 0.87 | 1.00 |
| Area IFJ1 (IFS PreCS) right | 28.80 | 80.97 | 0.86 | 0.80 | 0.92 |
| Area IFS4 (IFS) right | 28.49 | 80.01 | 0.87 | 0.81 | 0.93 |
| Area FG2 (FusG) right | 27.92 | 78.26 | 0.88 | 0.82 | 0.94 |
| Area IFS1 (IFS) right | 27.22 | 76.07 | 0.73 | 0.68 | 0.79 |
| Area hOc4lp (LOC) right | 24.99 | 68.89 | 0.71 | 0.66 | 0.77 |
| Area 45 (IFG) right | 24.05 | 65.80 | 0.52 | 0.48 | 0.56 |
| Area FG2 (FusG) left | 23.83 | 65.04 | 0.66 | 0.61 | 0.72 |
| Area FG1 (FusG) right | 22.65 | 61.09 | 0.63 | 0.57 | 0.68 |
| Area IFS4 (IFS) left | 21.36 | 56.69 | 0.67 | 0.61 | 0.73 |
| Area IFJ2 (IFS PreCS) right | 21.30 | 56.47 | 0.39 | 0.35 | 0.43 |
| Area IFJ2 (IFS PreCS) left | 21.29 | 56.43 | 0.50 | 0.45 | 0.54 |
| Area hOc2 (V2 18) left | 21.14 | 55.93 | 0.53 | 0.48 | 0.58 |
| Area IFS3 (IFS) right | 19.53 | 50.29 | 0.35 | 0.32 | 0.39 |
| Area IFS2 (IFS) left | 19.25 | 49.33 | 0.54 | 0.48 | 0.59 |
| Area hOc2 (V2 18) right | 19.19 | 49.10 | 0.52 | 0.46 | 0.57 |
| MF (Amygdala) left | 18.89 | 48.04 | 0.33 | 0.30 | 0.37 |
| Area hIP1 (IPS) right | 18.73 | 47.47 | 0.43 | 0.39 | 0.48 |
| Area 45 (IFG) left | 18.56 | 46.89 | 0.45 | 0.40 | 0.49 |
| Area IFJ1 (IFS PreCS) left | 18.56 | 46.89 | 0.52 | 0.47 | 0.58 |
| Area FG4 (FusG) left | 18.53 | 46.78 | 0.33 | 0.30 | 0.37 |
| Area hIP3 (IPS) left | 18.43 | 46.41 | 0.49 | 0.43 | 0.54 |
| Area hOc4la (LOC) right | 18.18 | 45.51 | 0.46 | 0.41 | 0.51 |
| Area IFS3 (IFS) left | 17.67 | 43.71 | 0.55 | 0.49 | 0.61 |
| Area Id8 (Insula) right | 17.61 | 43.50 | 0.45 | 0.40 | 0.50 |
| Area Id7 (Insula) right | 17.52 | 43.17 | 0.37 | 0.33 | 0.41 |
| MF (Amygdala) right | 17.50 | 43.10 | 0.35 | 0.31 | 0.39 |
| Area hIP1 (IPS) left | 17.17 | 41.93 | 0.41 | 0.36 | 0.45 |
| Area FG1 (FusG) left | 16.61 | 39.95 | 0.36 | 0.31 | 0.40 |
| Area hOc4lp (LOC) left | 16.53 | 39.65 | 0.37 | 0.33 | 0.42 |
| CM (Amygdala) right | 15.89 | 37.35 | 0.23 | 0.20 | 0.26 |
| Area Op9 (Frontal Operculum) right | 15.75 | 36.85 | 0.38 | 0.33 | 0.43 |
| Area 44 (IFG) left | 15.61 | 36.35 | 0.31 | 0.27 | 0.35 |
| Area hIP3 (IPS) right | 15.59 | 36.29 | 0.45 | 0.39 | 0.51 |
| SF (Amygdala) right | 15.23 | 35.00 | 0.27 | 0.23 | 0.30 |
| CM (Amygdala) left | 14.82 | 33.56 | 0.21 | 0.18 | 0.23 |
| Area Id8 (Insula) left | 14.61 | 32.80 | 0.33 | 0.29 | 0.38 |
| Area FG3 (FusG) right | 14.54 | 32.56 | 0.28 | 0.24 | 0.32 |
| Area hIP6 (IPS) right | 14.42 | 32.12 | 0.45 | 0.38 | 0.51 |
| Area IFS1 (IFS) left | 14.38 | 31.99 | 0.37 | 0.32 | 0.42 |
| SF (Amygdala) left | 13.98 | 30.55 | 0.23 | 0.20 | 0.26 |
| LB (Amygdala) right | 13.94 | 30.43 | 0.20 | 0.17 | 0.23 |
| Area hOc1 (V1 17 CalcS) right | 13.74 | 29.72 | 0.35 | 0.30 | 0.40 |
| Area Op9 (Frontal Operculum) left | 13.59 | 29.19 | 0.33 | 0.28 | 0.38 |
| Area Id7 (Insula) left | 13.19 | 27.76 | 0.26 | 0.22 | 0.30 |
| Area hIP6 (IPS) left | 13.02 | 27.20 | 0.33 | 0.28 | 0.37 |
| VTM (Amygdala) left | 12.73 | 26.20 | 0.17 | 0.15 | 0.20 |
| CGL (Metathalamus) right | 12.50 | 25.38 | 0.19 | 0.16 | 0.22 |
| Ch 4 (Basal Forebrain) right | 12.45 | 25.20 | 0.14 | 0.12 | 0.17 |
| Frontal II (GapMap) right | 12.23 | 24.46 | 0.16 | 0.14 | 0.19 |
| Frontal to Temporal I (GapMap) left | 11.93 | 23.44 | 0.30 | 0.25 | 0.35 |
| Area Fo7 (OFC) right | 11.81 | 23.02 | 0.26 | 0.22 | 0.31 |
| Frontal to Temporal I (GapMap) right | 11.62 | 22.38 | 0.29 | 0.24 | 0.34 |
| HATA (Hippocampus) left | 11.48 | 21.89 | 0.21 | 0.17 | 0.24 |
| VTM (Amygdala) right | 11.47 | 21.85 | 0.17 | 0.14 | 0.20 |
| HATA (Hippocampus) right | 11.46 | 21.83 | 0.21 | 0.18 | 0.25 |
| Area Op8 (Frontal Operculum) left | 11.38 | 21.57 | 0.21 | 0.17 | 0.24 |
| CGM (Metathalamus) right | 11.37 | 21.54 | 0.18 | 0.15 | 0.21 |
| Area hIP2 (IPS) right | 10.90 | 19.97 | 0.27 | 0.22 | 0.32 |
| Frontal II (GapMap) left | 10.73 | 19.40 | 0.17 | 0.14 | 0.21 |
| Area Fo6 (OFC) left | 10.52 | 18.72 | 0.28 | 0.23 | 0.33 |
| CGL (Metathalamus) left | 10.45 | 18.48 | 0.15 | 0.12 | 0.18 |
| Area Fo4 (OFC) right | 10.21 | 17.70 | 0.26 | 0.21 | 0.31 |
| LB (Amygdala) left | 10.13 | 17.45 | 0.15 | 0.12 | 0.18 |
| Fastigial Nucleus (Cerebellum) right | 10.08 | 17.27 | 0.14 | 0.12 | 0.17 |
| Area hIP8 (IPS) right | 10.04 | 17.14 | 0.26 | 0.21 | 0.31 |
| Ch 4 (Basal Forebrain) left | 10.03 | 17.12 | 0.13 | 0.11 | 0.16 |
| Area hOc4la (LOC) left | 9.97 | 16.92 | 0.22 | 0.18 | 0.27 |
| Area hOc1 (V1 17 CalcS) left | 9.91 | 16.76 | 0.24 | 0.20 | 0.29 |
| Area Fo6 (OFC) right | 9.86 | 16.58 | 0.22 | 0.18 | 0.27 |
| CGM (Metathalamus) left | 9.84 | 16.51 | 0.16 | 0.13 | 0.20 |
| Area hIP4 (IPS) right | 9.79 | 16.36 | 0.25 | 0.20 | 0.30 |
| Area 44 (IFG) right | 9.55 | 15.59 | 0.18 | 0.14 | 0.21 |
| Area Fo3 (OFC) right | 9.43 | 15.23 | 0.16 | 0.13 | 0.20 |
| Area hOc5 (LOC) right | 9.27 | 14.72 | 0.24 | 0.19 | 0.29 |
| Area Fo5 (OFC) right | 9.23 | 14.60 | 0.27 | 0.21 | 0.33 |
| Interposed Nucleus (Cerebellum) right | 9.15 | 14.35 | 0.12 | 0.09 | 0.14 |
| Fastigial Nucleus (Cerebellum) left | 8.90 | 13.60 | 0.12 | 0.09 | 0.14 |
| Area Op8 (Frontal Operculum) right | 8.85 | 13.46 | 0.18 | 0.14 | 0.22 |
| Area Ph1 (PhG) right | 8.66 | 12.88 | 0.15 | 0.12 | 0.19 |
| Area 6d3 (SFS) right | 8.57 | 12.61 | 0.12 | 0.09 | 0.14 |
| Area 6ma (preSMA mesial SFG) left | 8.53 | 12.50 | 0.14 | 0.11 | 0.18 |
| Area hIP2 (IPS) left | 8.41 | 12.15 | 0.20 | 0.16 | 0.25 |
| Area hIP7 (IPS) right | 8.40 | 12.12 | 0.20 | 0.15 | 0.24 |
| BST (Bed Nucleus) right | 8.32 | 11.89 | 0.13 | 0.10 | 0.17 |
| Frontal I (GapMap) right | 8.02 | 11.03 | 0.16 | 0.12 | 0.20 |
| Area Id10 (Insula) right | 7.86 | 10.57 | 0.15 | 0.11 | 0.18 |
| Area 6ma (preSMA mesial SFG) right | 7.76 | 10.28 | 0.13 | 0.10 | 0.17 |
| Frontal to Temporal II (GapMap) right | 7.76 | 10.28 | 0.11 | 0.08 | 0.14 |
| Entorhinal Cortex right | 7.56 | 9.72 | 0.13 | 0.10 | 0.16 |
| BST (Bed Nucleus) left | 7.40 | 9.28 | 0.12 | 0.08 | 0.15 |
| Area 8v1 (MFG) right | 7.25 | 8.90 | 0.15 | 0.11 | 0.19 |
| Area Fo5 (OFC) left | 6.64 | 7.30 | 0.20 | 0.14 | 0.26 |
| Area hIP5 (IPS) left | 6.36 | 6.60 | 0.15 | 0.11 | 0.20 |
| Area Fo4 (OFC) left | 6.19 | 6.19 | 0.18 | 0.12 | 0.24 |
| Area hIP7 (IPS) left | 6.15 | 6.08 | 0.16 | 0.11 | 0.21 |
| Area hIP8 (IPS) left | 5.90 | 5.50 | 0.16 | 0.11 | 0.21 |
| Temporal to Parietal (GapMap) right | 5.84 | 5.35 | 0.07 | 0.05 | 0.10 |
| Frontal I (GapMap) left | 5.75 | 5.14 | 0.13 | 0.09 | 0.18 |
| Area Fo7 (OFC) left | 5.70 | 5.03 | 0.15 | 0.10 | 0.20 |
| Area FG3 (FusG) left | 5.69 | 5.02 | 0.08 | 0.05 | 0.11 |
| Ch 123 (Basal Forebrain) right | 5.53 | 4.64 | 0.09 | 0.05 | 0.12 |
| Area Fo3 (OFC) left | 5.48 | 4.54 | 0.10 | 0.06 | 0.14 |
| Frontal to Temporal II (GapMap) left | 5.41 | 4.38 | 0.07 | 0.04 | 0.09 |
| Area 7P (SPL) right | 5.34 | 4.24 | 0.21 | 0.13 | 0.29 |
| Area Id10 (Insula) left | 5.26 | 4.07 | 0.10 | 0.06 | 0.14 |
| Ch 123 (Basal Forebrain) left | 5.16 | 3.85 | 0.08 | 0.05 | 0.11 |
| Area 7A (SPL) right | 4.96 | 3.44 | 0.13 | 0.08 | 0.19 |
| Area 6d3 (SFS) left | 4.77 | 3.05 | 0.07 | 0.04 | 0.10 |
| Area 6d2 (PreCG) left | 4.63 | 2.79 | 0.08 | 0.05 | 0.12 |
| Area hOc3d (Cuneus) right | 4.55 | 2.61 | 0.11 | 0.06 | 0.16 |
| Area hOc5 (LOC) left | 4.48 | 2.49 | 0.10 | 0.06 | 0.15 |
| Ventral Dentate Nucleus (Cerebellum) right | 4.46 | 2.46 | 0.05 | 0.03 | 0.08 |
| Area Id6 (Insula) left | 4.44 | 2.41 | 0.09 | 0.05 | 0.13 |
| Entorhinal Cortex left | 4.28 | 2.11 | 0.07 | 0.04 | 0.10 |
| Interposed Nucleus (Cerebellum) left | 4.27 | 2.10 | 0.05 | 0.03 | 0.08 |
| HC Subiculum (Hippocampus) right | 4.08 | 1.75 | 0.07 | 0.03 | 0.10 |
| Area hIP4 (IPS) left | 3.99 | 1.60 | 0.12 | 0.06 | 0.18 |

**Supplementary Table 4.** Brain activation to shapes over faces (*p* < 0.05, Bonferroni corrected).

| *Region* | *t_269_* | *-log_10_ p* | *Contrast estimate* | *95 CI lower* | *95 CI upper* |
| --- | --- | --- | --- | --- | --- |
| Area OP1 (POperc) right | 13.73 | 29.69 | 0.25 | 0.22 | 0.29 |
| Area TE 3 (STG) right | 13.53 | 28.98 | 0.21 | 0.18 | 0.25 |
| Area TE 1.2 (HESCHL) right | 13.34 | 28.32 | 0.25 | 0.21 | 0.28 |
| Area TE 3 (STG) left | 12.40 | 25.05 | 0.28 | 0.23 | 0.32 |
| Area TE 2.1 (STG) right | 11.72 | 22.72 | 0.23 | 0.19 | 0.27 |
| Area OP4 (POperc) right | 11.72 | 22.70 | 0.19 | 0.16 | 0.22 |
| Area Op6 (Frontal Operculum) right | 11.60 | 22.32 | 0.26 | 0.22 | 0.31 |
| Area PFcm (IPL) left | 11.14 | 20.77 | 0.20 | 0.17 | 0.24 |
| Area TE 2.2 (STG) left | 10.94 | 20.08 | 0.21 | 0.17 | 0.24 |
| Area PFop (IPL) left | 10.89 | 19.92 | 0.20 | 0.17 | 0.24 |
| Area Fp2 (FPole) left | 10.83 | 19.74 | 0.40 | 0.32 | 0.47 |
| Area Id2 (Insula) right | 10.78 | 19.56 | 0.20 | 0.16 | 0.24 |
| Area TE 2.2 (STG) right | 10.71 | 19.33 | 0.16 | 0.13 | 0.19 |
| Area s32 (sACC) right | 10.59 | 18.92 | 0.23 | 0.19 | 0.28 |
| Area TE 1.0 (HESCHL) right | 10.28 | 17.92 | 0.20 | 0.16 | 0.24 |
| Area Id1 (Insula) left | 10.03 | 17.11 | 0.18 | 0.15 | 0.22 |
| Area PFcm (IPL) right | 9.93 | 16.81 | 0.14 | 0.11 | 0.17 |
| Area Ph2 (PhG) left | 9.92 | 16.78 | 0.14 | 0.12 | 0.17 |
| Area Op5 (Frontal Operculum) right | 9.65 | 15.91 | 0.15 | 0.12 | 0.18 |
| Area TE 2.1 (STG) left | 9.48 | 15.40 | 0.19 | 0.15 | 0.23 |
| Area s32 (sACC) left | 9.47 | 15.36 | 0.20 | 0.16 | 0.24 |
| Area TE 1.2 (HESCHL) left | 9.43 | 15.22 | 0.19 | 0.15 | 0.22 |
| Area Id1 (Insula) right | 9.42 | 15.21 | 0.15 | 0.12 | 0.18 |
| Area Ig2 (Insula) right | 9.42 | 15.21 | 0.15 | 0.11 | 0.18 |
| Area PGp (IPL) left | 9.12 | 14.28 | 0.20 | 0.16 | 0.24 |
| Area TI (STG) left | 9.10 | 14.20 | 0.19 | 0.15 | 0.23 |
| Area 5Ci (SPL) right | 9.04 | 14.04 | 0.15 | 0.12 | 0.18 |
| Area TE 1.1 (HESCHL) right | 8.93 | 13.68 | 0.13 | 0.10 | 0.16 |
| Area OP1 (POperc) left | 8.89 | 13.58 | 0.16 | 0.12 | 0.19 |
| Area Id3 (Insula) right | 8.76 | 13.18 | 0.16 | 0.13 | 0.20 |
| Area TeI (STG) left | 8.60 | 12.72 | 0.19 | 0.15 | 0.24 |
| Area 5Ci (SPL) left | 8.60 | 12.70 | 0.12 | 0.09 | 0.14 |
| Area TPJ (STG SMG) left | 8.51 | 12.45 | 0.17 | 0.13 | 0.21 |
| Area TE 1.0 (HESCHL) left | 8.51 | 12.44 | 0.16 | 0.13 | 0.20 |
| Area Id3 (Insula) left | 8.45 | 12.26 | 0.17 | 0.13 | 0.21 |
| Area Ig2 (Insula) left | 8.42 | 12.18 | 0.14 | 0.11 | 0.18 |
| Area PFop (IPL) right | 8.20 | 11.53 | 0.14 | 0.10 | 0.17 |
| Area p24c (pACC) left | 8.15 | 11.39 | 0.23 | 0.17 | 0.29 |
| Area TPJ (STG SMG) right | 8.09 | 11.20 | 0.16 | 0.12 | 0.19 |
| Area Id2 (Insula) left | 7.94 | 10.79 | 0.15 | 0.11 | 0.19 |
| Area OP3 (POperc) right | 7.75 | 10.26 | 0.11 | 0.08 | 0.14 |
| Area TE 1.1 (HESCHL) left | 7.72 | 10.17 | 0.14 | 0.11 | 0.18 |
| Area Fp2 (FPole) right | 7.69 | 10.09 | 0.25 | 0.19 | 0.32 |
| Area OP4 (POperc) left | 7.59 | 9.81 | 0.14 | 0.10 | 0.17 |
| Area CoS1 (CoS) left | 7.15 | 8.63 | 0.11 | 0.08 | 0.14 |
| Area p24ab (pACC) right | 6.99 | 8.19 | 0.16 | 0.11 | 0.20 |
| Area STS1 (STS) left | 6.95 | 8.10 | 0.11 | 0.08 | 0.14 |
| CA3 (Hippocampus) left | 6.70 | 7.44 | 0.09 | 0.06 | 0.11 |
| Area OP2 (POperc) right | 6.68 | 7.41 | 0.09 | 0.06 | 0.11 |
| Area TI (STG) right | 6.57 | 7.12 | 0.13 | 0.09 | 0.17 |
| Area p24ab (pACC) left | 6.55 | 7.07 | 0.14 | 0.09 | 0.18 |
| Area 8d2 (SFG) left | 6.40 | 6.70 | 0.11 | 0.08 | 0.15 |
| Area Ig1 (Insula) right | 6.26 | 6.36 | 0.09 | 0.06 | 0.12 |
| Area Ia1 (Insula) right | 6.21 | 6.24 | 0.12 | 0.08 | 0.16 |
| Area 3b (PostCG) right | 6.01 | 5.75 | 0.07 | 0.05 | 0.10 |
| Area p32 (pACC) left | 5.84 | 5.35 | 0.16 | 0.11 | 0.21 |
| Area Id5 (Insula) right | 5.84 | 5.35 | 0.11 | 0.07 | 0.14 |
| Area Ig3 (Insula) right | 5.81 | 5.28 | 0.08 | 0.06 | 0.11 |
| Area 3a (PostCG) right | 5.80 | 5.27 | 0.08 | 0.05 | 0.10 |
| Area Ig1 (Insula) left | 5.68 | 4.99 | 0.09 | 0.06 | 0.12 |
| Area Ia1 (Insula) left | 5.68 | 4.98 | 0.11 | 0.07 | 0.15 |
| Frontal to Occipital (GapMap) left | 5.46 | 4.50 | 0.08 | 0.05 | 0.11 |
| Area PGp (IPL) right | 5.42 | 4.42 | 0.13 | 0.08 | 0.18 |
| Area Ia2 (Insula) right | 5.30 | 4.15 | 0.16 | 0.10 | 0.22 |
| Area STS2 (STS) left | 5.19 | 3.91 | 0.08 | 0.05 | 0.12 |
| Area Id4 (Insula) right | 5.13 | 3.78 | 0.08 | 0.05 | 0.11 |
| CA2 (Hippocampus) left | 4.98 | 3.48 | 0.07 | 0.04 | 0.10 |
| Area Op5 (Frontal Operculum) left | 4.95 | 3.41 | 0.09 | 0.05 | 0.12 |
| Area 6mp (SMA mesial SFG) right | 4.90 | 3.32 | 0.07 | 0.04 | 0.09 |
| Area 5L (SPL) left | 4.90 | 3.31 | 0.10 | 0.06 | 0.14 |
| Area p32 (pACC) right | 4.89 | 3.30 | 0.12 | 0.07 | 0.17 |
| Area 4p (PreCG) right | 4.86 | 3.23 | 0.07 | 0.04 | 0.10 |
| Area OP3 (POperc) left | 4.75 | 3.01 | 0.07 | 0.04 | 0.10 |
| Area s24 (sACC) right | 4.70 | 2.90 | 0.07 | 0.04 | 0.10 |
| Area Ig3 (Insula) left | 4.66 | 2.82 | 0.07 | 0.04 | 0.10 |
| HC Parasubiculum (Hippocampus) right | 4.56 | 2.63 | 0.07 | 0.04 | 0.11 |
| Area OP2 (POperc) left | 4.54 | 2.60 | 0.07 | 0.04 | 0.10 |
| Area 1 (PostCG) right | 4.34 | 2.22 | 0.07 | 0.04 | 0.10 |
| Area Ia2 (Insula) left | 4.23 | 2.03 | 0.11 | 0.06 | 0.16 |
| Area Id5 (Insula) left | 4.20 | 1.96 | 0.08 | 0.04 | 0.12 |
| Area 5M (SPL) left | 3.91 | 1.46 | 0.07 | 0.04 | 0.11 |
| Area PFt (IPL) right | 3.82 | 1.32 | 0.06 | 0.03 | 0.09 |
| Area 6mp (SMA mesial SFG) left | 3.82 | 1.32 | 0.05 | 0.02 | 0.08 |

**Supplementary Table 5.** Results from the voxel-based analysis. Area of local maximum was extracted using SPM Anatomy toolbox v2.2. Brain areas activated by the faces over shapes (*p* < 0.05 family-wise error corrected). R = right hemisphere, L = left hemisphere.

|  |  |  | MNI | | |  |  |
| --- | --- | --- | --- | --- | --- | --- | --- |
| Contrast | Area of local maximum | *T* | X | Y | Z | Cluster | Number of voxels |
| Faces over shapes | L Inferior Occipital Gyrus | 39.28 | -16 | -100 | -4 | 1 | 46648 |
|  | R Inferior Occipital Gyrus | 36.57 | 28 | -90 | -10 |  |  |
|  | R Cerebelum (VI) | 34.38 | 42 | -54 | -20 |  |  |
|  | R Superior Medial Gyrus | 24.78 | 4 | 16 | 50 | 2 | 3301 |
|  | L Posterior-Medial Frontal | 5.29 | -10 | 8 | 70 |  |  |
|  | L Middle Temporal Gyrus | 8.42 | -54 | -50 | 8 | 3 | 318 |
|  | R Medial Temporal Pole | 5.80 | 42 | 16 | -32 | 4 | 15 |
|  | L Inferior Temporal Gyrus | 4.74 | -36 | -14 | -28 | 5 | 1 |

**Supplementary Figure 1**. Genetic influence on brain areas showing greater responses to shapes than faces. Green areas denote combined brain regions from the Julich brain atlas in which genetic influence was demonstrated for responses to shapes over faces. All statistics are Bonferroni-corrected at *p* < 0.05.


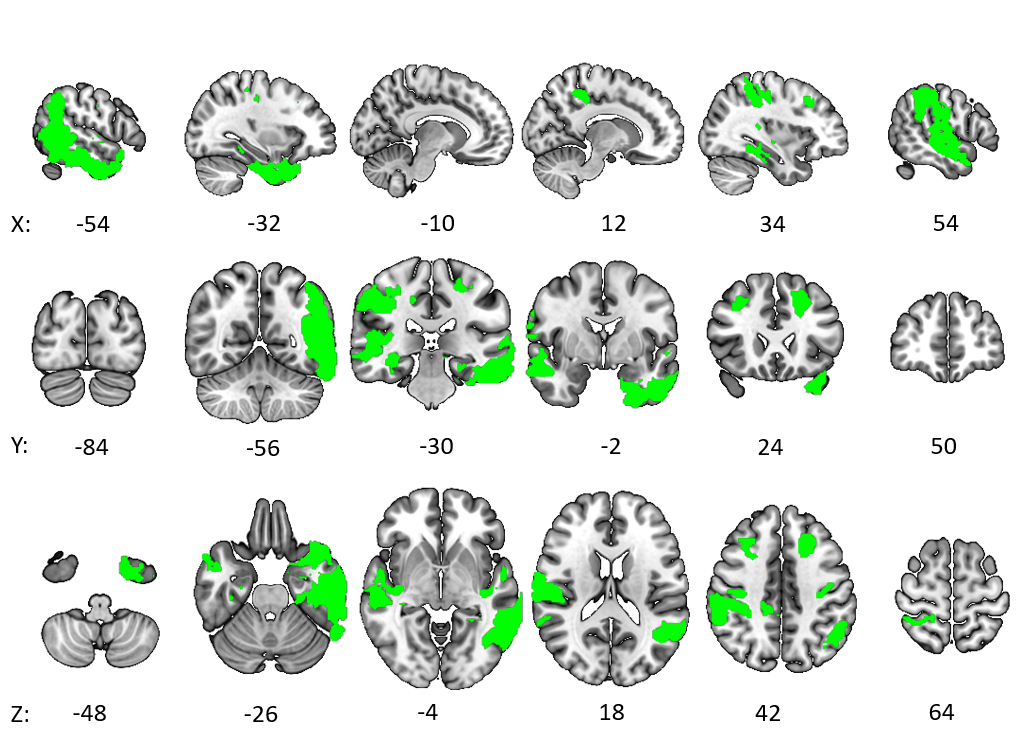

Supplement: Supplementary file 1 — Supplemental material [file 41398_2026_4240_MOESM1_ESM.docx]
